# Spatial–temporal evolution and spatial spillover of the green efficiency of urban construction land in the Yangtze River Economic Belt, China

**DOI:** 10.1038/s41598-023-41621-4

**Published:** 2023-09-01

**Authors:** Jialiang Zhou

**Affiliations:** School of Economics and Trade, Fujian Jiangxia University, Fuzhou, 350108 China

**Keywords:** Ecology, Environmental sciences

## Abstract

There are urgent ecological and environmental problems in the process of the utilization of urban construction land, promoting green utilization of construction land is conducive to urban sustainable development and high-quality economic development. Based on the panel data of 108 prefecture-level and above cities in the Yangtze River Economic Belt, China from 2003 to 2017, this paper uses the super-efficiency SBM model to measure the green efficiency of urban construction land (GEUCL), analyzes its spatial–temporal evolution characteristics, and constructs the spatial autoregressive model to study its spatial spillover effects from the perspective of urban hierarchy. It is found that, in terms of temporal variation, the average efficiency value shows a fluctuating upward trend during the study period, rising from 0.27 in 2003 to 0.39 in 2017, the cumulative growth rate is 44.44%, with an average annual growth rate of 3.14%. In terms of spatial distribution characteristics, during the study period, the number of medium-efficiency and high-efficiency cities increases significantly, while the number of low-efficiency cities decreases sharply; high-efficiency cities always present scattered distribution, while medium-efficiency cities change from scattered distribution to agglomeration distribution. In addition, GEUCL has significantly positive spatial spillover effects between neighboring cities of different grades and between neighboring cities of the same grade, among them, the increase of GEUCL in higher-grade cities has significantly positive spatial spillover effects on that in adjacent lower-grade cities; the increase of GEUCL in lower-grade cities has significantly positive spatial spillover effects on that in neighboring higher-grade cities; GEUCL has significantly positive spatial spillover effects between neighboring cities of the same grade.

## Introduction

In recent years, the rapid development of global urbanization has greatly changed people's material life, but the further development of cities and urban governance are faced with many challenges that restrict sustainable development, such as tightening resource constraints, ecosystem degradation, and environmental pollution. In order to solve the difficulties encountered in the process of global urbanization, we must pay attention to the key role of the ecosystem, and realize the positive interaction between economic development and environmental protection through green development, as well as the harmonious coexistence of man and nature. As we all know, green development is a mode of economic growth and social development aiming at efficiency, harmony and sustainability, and a new development mode that takes environmental protection as an important pillar to achieve sustainable development. As the basic carrier of production activities and urban development, land resources play a decisive role in the process of global economic development. It can be said that the use of land resources is accompanied by the whole process of urban economic development and urbanization. It is undeniable that in terms of land use, the world is also facing difficulties such as resource shortage, environmental pollution and land ecosystem deterioration. Since the reform and opening up, China has made remarkable achievements in economic development by virtue of the development and utilization of urban construction land. However, with the rapid progress of urbanization and industrialization, ecological and environmental problems arising in the use of urban construction land in China have become increasingly serious. Under the theme of green development, how to break the existing path dependence of the utilization of urban construction land and realize green utilization is an important issue that must be faced directly. As a matter of fact, it is important and urgent to study the green efficiency of urban construction land (GEUCL) as the evaluation standard and performance index of green utilization of construction land.

In this paper, the green efficiency of urban construction land is defined as the maximum economic benefit obtained from the minimum resource, economic input and environmental pollution cost in the process of construction land utilization under certain production technology conditions. It provides a solution for the evaluation of green utilization of urban construction land, which is conducive to more efficient use and management of land^1^. The research on the green efficiency of urban construction land originated from the exploration of the measurement of urban land use efficiency. According to the different research purposes and research methods, the measurement methods of urban land use efficiency were different. Among them, the simplest method was to adopt the output per unit of land to measure ULGUE^2^. This kind of method had certain limitations and ignored that land use is a complex system. With the deepening of research, many literatures used input–output model to describe the input–output relationship in the process of land use, so as to measure urban land use efficiency^3, 4^, this method is more scientific and reasonable, and has been widely used. It is worth noting that some scholars also use per-capita build-up area^5^, urban density^6^, the ratio of land consumption rate to population growth rate^7–9^ and other relevant variables to act as proxies for urban land use efficiency.

In order to provide more detailed reference for the formulation of land use policy, scholars focused on exploring its influencing factors on the basis of measuring urban land use efficiency, and found that the factors affecting urban land use efficiency were different in different regions. In the tourist destination, Davos of the Swiss Alps, the driving factors of urban land use efficiency included the economic impact of tourists, occupancy intensity and the density of beds per area covered by residential buildings and hotels^10^. In Southern Europe, mixed land uses, multiple-use buildings, vertical profile were the most important factors affecting urban land use efficiency^11^. In Lombardy of Italy, urban size could affect urban land use efficiency, the larger the urban size, the higher the urban land use efficiency^5^. In Addis Ababa of Ethiopia, land use efficiency was low in most cities, and land hoarding and land use fragmentation were the main factors affecting land use efficiency^12, 13^. In Vietnam, changes in land use rights could affect land use efficiency^14^. In Netherlands, Belgium and Poland, urban planning was an important factor influencing urban land use efficiency^15^. In addition, Chakraborty et al.^6^ conducted a study using 466 global cities with more than one million population as a sample, and found that in the Global North, inward expansion of cities decreases urban land use efficiency, while in the Global South, the result is the opposite. Masini et al.^16^ discussed urban land use efficiency in 417 metropolitan regions from 27 European countries, it was found that there are higher land use efficiency in richer cities. Langpap et al.^17^ pointed out that land use policies with incentive properties can significantly promote the improvement of land use efficiency. Deshmukh et al.^18^ found that taking measures to optimize allocation in the process of urban land resource development and utilization can improve land use efficiency.

With the increasing attention paid to environmental protection and green development, some scholars took environmental constraints into consideration when studying urban land use efficiency, and conducted a large number of effective studies on the green use efficiency of urban land. In terms of efficiency measurement, existing studies adopted SBM model^19, 20^, super-efficiency SBM model^21–25^, global non-radial directional distance function^26^, EBM model^27^ to measure the green use efficiency of urban land. It is worth noting that although there were differences in various measurement methods, environmental pollution generated in the process of land use was regarded as an undesirable output. In terms of spatial–temporal pattern and dynamic evolution characteristics, based on the objective facts of regional differences in industrial policies and resource endowments, existing studies discussed the spatial–temporal pattern^22, 28^, spatial correlation^26^, spatial agglomeration characteristics^20, 28^ and spatial heterogeneity^21, 29^ of the green use efficiency of urban land, the main purpose of this research is to grasp and predict the change trend of the green use efficiency of urban land. In terms of influencing factors, scholars investigated the impact of urban agglomeration construction^30^, smart city construction^23^, multi-center development^19^, economic development level and industrial structure upgrading^28, 31^, land finance^32^, location-oriented policies^33^, low-carbon city pilot policies^34, 35^, new urbanization^24^ and other factors on the green use efficiency of urban land, the exploration of driving factors and hindering factors provides a reasonable solution for promoting improvement of GEUCL.

With the deepening of research, scholars tended to refine their research on the green use efficiency of urban land, and began to pay attention to the green use efficiency of urban land at the level of urban construction land, namely GEUCL. Wang et al.^36^ measured the GEUCL of China and the United States by taking carbon emissions as the undesired output, and found that the efficiency values in both countries were at a relatively low level. Gao et al.^37^ took the Beijing-Tianjin-Hebei region of China as the research area to explore GEUCL under the constraint of carbon emissions, and found that GEUCL presented the trend of “first decreasing and then rising” and the northeast-southwest distribution. Liang and Chen^38^ measured GEUCL under environmental constraints in Fujian Province, China, and confirmed that efficiency values in different regions were significantly different, presenting a spatial pattern that the efficiency values in coastal areas are higher than those in inland areas, and showing a significant cluster effect. Li et al.^39^ also took Fujian Province of China as the research area to measure GEUCL under the constraint of carbon emissions, and found that in general, GEUCL showed an evolution characteristic of “first rising, then falling and then rising”, in terms of spatial differentiation, GEUCL presented a steady growth trend in coastal economically developed cities, while a fluctuating upward trend in other cities.

Based on the above literature review, this paper believes that the existing relevant literature has laid a solid foundation for this study, and also provided a reference basis for improving urban land governance and improving urban green development performance. However, the existing literature mainly focused on the measurement and spatial–temporal evolution characteristics of GEUCL, and rarely involved the spatial spillover effects of GEUCL. Under the trend of regional economic integration and the background of increasingly close economic ties between cities, the utilization of urban construction land will inevitably be affected by surrounding cities, and the impact between cities of different grades may be different. Research on GEUCL, reasonably evaluate the green efficiency of urban construction land under environmental constraints, and explore the spatial spillover effects of GEUCL between cities of different grades on the basis of analyzing the spatial–temporal evolution characteristics of GEUCL, which is of great theoretical significance and practical value for breaking regional resource and environment constraints, reconciling the contradiction between regional economic development and ecological environment, guiding the green, efficient and sustainable utilization of urban construction land, and jointly promoting the green utilization of regional urban construction land and regional sustainable development. In addition, this study is helpful to enrich the literature in the field of green efficiency of urban construction land and expand the theoretical content of land use and green development.

Firstly, Based on the panel data of 108 prefecture-level and above cities in the Yangtze River Economic Belt, China from 2003 to 2017, this paper uses the super-efficiency SBM model containing unexpected outputs to measure the green efficiency of urban construction land. Secondly, the spatial–temporal evolution characteristics of GEUCL are analyzed based on the measurement results. Finally, from the perspective of urban hierarchy, this paper constructs a spatial autoregressive model to explore the spatial interaction of GEUCL, and analyzes the spatial spillover effects of GEUCL between cities of different grades. The rest of this paper is arranged as follows: the second part is materials and methods, the third part is results, and the fourth part is discussion, the fifth part is conclusions.

## Materials and methods

### Study area

**Figure 1:**
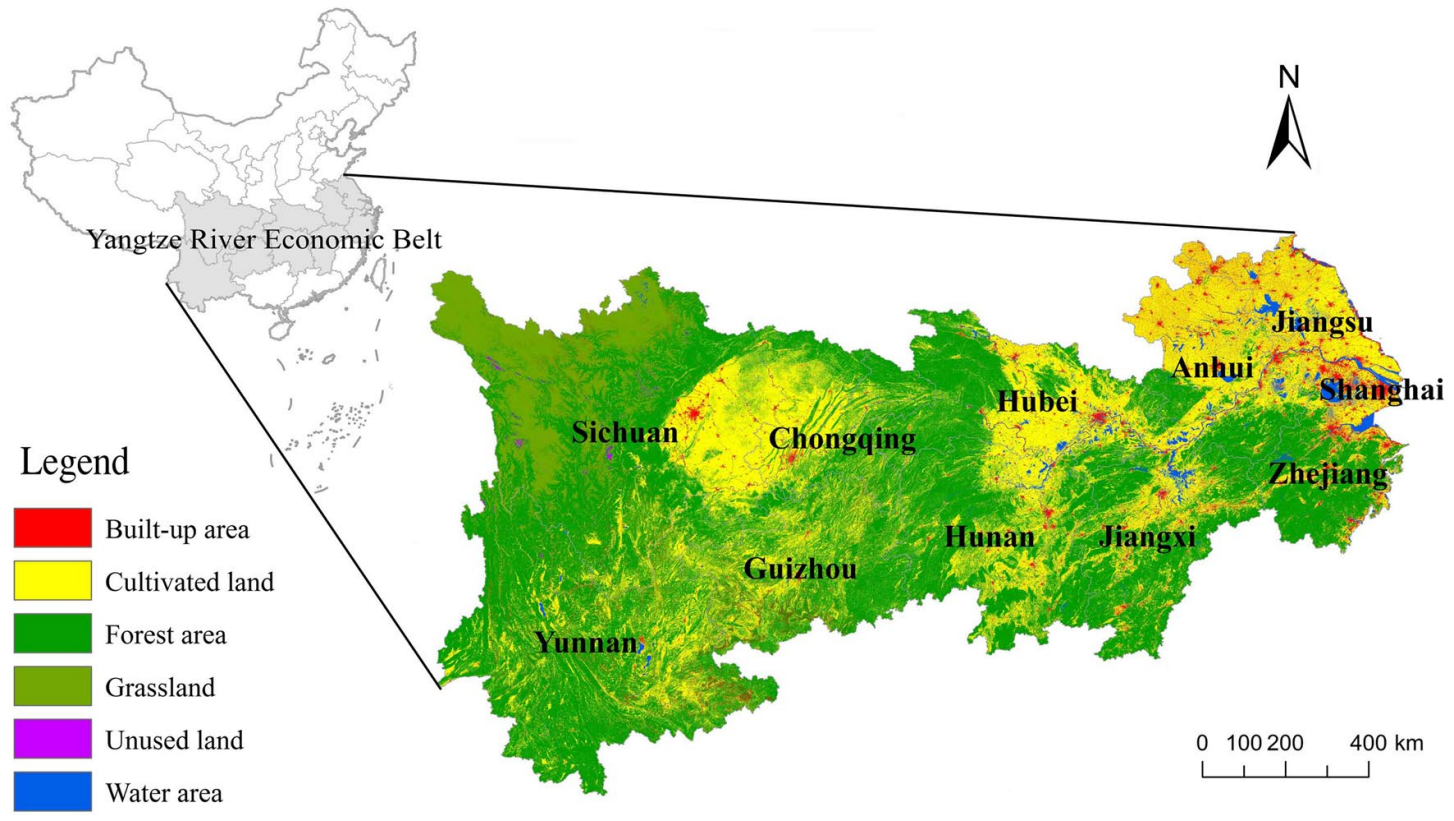
The geographical position of the Yangtze River Economic Belt, China.

China’s Yangtze River Economic Belt is an area through which the main stream of the Yangtze River flows (as shown in Fig. 1), and spans the eastern, central and western parts of China, is major national strategic development area in China. Since the reform and opening up, the Yangtze River Economic Belt has developed into one of the regions with the strongest comprehensive strength and the greatest strategic support in China, relying on its advantages in transportation, resources, industries and market. The Yangtze River Economic Belt includes 126 cities at prefecture or above level, covering 11 provincial-level administrative regions, including Shanghai, Jiangsu, Zhejiang, Anhui, Jiangxi, Hubei, Hunan, Chongqing, Sichuan, Yunnan and Guizhou. It covers an area of about 2.05 million square kilometers, accounting for 21.4 percent of China’s total area. In 2018, the population in the Yangtze River Economic Belt was about 599 million, accounting for 42.9% of China’s total population, and gross regional product was about 40.3 trillion Yuan, accounting for 44.1 percent of China’ GDP. According to the features of the Yangtze River channel and the topography of the basin, the Yangtze Economic Belt is divided into the upstream area, midstream area and downstream area. The upstream area includes 47 cities, covering an area of 1.1374 million square kilometers, accounting for 55.4% of the total area of the Yangtze River Economic Belt; the midstream area includes 38 cities, covering an area of 564,600 square kilometers, accounting for 27.5 percent of the total area of the Yangtze River Economic Belt; the downstream area includes 41 cities, covering an area of about 350,300 square kilometers, accounting for 17.1 percent of the total area of the Yangtze River Economic Belt. In recent years, the ecological and environmental situation in the Yangtze River Economic Belt has become grim. Therefore, the China’s government has put forward the strategic orientation of giving priority to ecology and green development, which is committed to building a green ecological corridor along the Yangtze River. Studying GEUCL in Yangtze River Economic Belt of China is of great practical significance for promoting the green utilization of urban construction land in this region and promoting the coordinated and sustainable development of this region.

### Data sources

This paper takes Yangtze River Economic Belt as the research area, which contains 126 cities at prefecture and above level. Because the data is unavailable or missing seriously in Enshi Prefecture of Hubei Province, Xiangxi Prefecture of Hunan Province, Aba Prefecture, Ganzi Prefecture and Liangshan Prefecture of Sichuan Province, Bijie City, Tongren City, Qiandongnan Prefecture, Qiandongnan Prefecture, Qiandxinan Prefecture of Guizhou Province, Chuxiong Prefecture, Honghe Prefecture, Diqing Prefecture, Wenshan Prefecture, Xishuangbanna Prefecture, Dali Prefecture, Dehong Prefecture and Nujiang Prefecture of Yunnan Province, these 18 prefectures or cities are excluded, and the number of final sample cities is 108, including 31 cities in the upstream area, 36 cities in the midstream area, and 41 cities in the downstream area. Therefore, the panel data of 108 cities at prefecture and above level in China's Yangtze River Economic Belt from 2003 to 2017 are selected as research samples in this paper. The original data come from China City Statistical Yearbook, China City Construction Statistical Yearbook and statistical yearbooks of provinces involved in Yangtze River Economic Belt. For missing data, interpolation method is used to supplement data in this paper.

### Methods

#### Super-efficiency SBM model for measuring GEUCL

DEA model is a common method to measure efficiency, but the traditional DEA model does not solve the slack problem of input–output variables, and does not take the unexpected output generated in the production process into account, so there will be a bias in measuring the efficiency of economic variables with undesired output. In order to correct the traditional DEA model, Tone proposed a non-radial and non-angular slack-based model (SBM), which can effectively solve the problems of the slack of variables and undesired outputs^40^. However, this model cannot reorder multiple effective decision making units with efficiency value of 1, which will affect the accuracy of efficiency value to some extent. Therefore, Tone improved the original SBM model, combined the super-efficiency model with the SBM model, proposed the super-efficiency SBM model, which can further decompose the decision making units with efficiency value of 1, and enable reordering effective decision making units^41^. In this paper, the green efficiency of urban construction land is defined as the input–output relationship of land use that takes environmental pollution caused by the utilization of urban construction land into consideration, therefore, this paper draws on the research method of Tone and uses the super-efficiency SBM model containing unexpected output to measure GEUCL^41^. The model is constructed as follows:

Suppose there are N decision making units (DMUs), L kinds of input factors, D kinds of expected outputs, and V kinds of unexpected outputs. The vector of input factors is defined as *X* = [*x*_*1*_, … , *x*_*n*_], the vector of expected outputs is defined as *Y*^*g*^ = [*yg 1*, … , *yg n*], and the vector of unexpected outputs is defined as *Y*^*b*^ = [*yb 1*, … , *yb n*], then the production possibility set is:

1 $$P\left( {x,y^{g} ,y^{{\text{b}}} } \right){ = }\left\{ {\left( {x,y^{g} ,y^{{\text{b}}} } \right)\left| {\sum\limits_{n = 1}^{N} {z_{n} x_{n} \le x,\sum\limits_{n = 1}^{N} {z_{n} y_{{_{n} }}^{g} \rm { \ge }y^{g} ,} } } \right.\sum\limits_{n = 1}^{N} {z_{n} y_{{_{n} }}^{b} \le y^{b} } } \right\}$$

Then the linear programming formula of the super-efficiency SBM model is as follows:

2 $$\begin{aligned} \rho & \rm { = }\min \frac{{\frac{1}{L}\sum\limits_{{{\text{l}}\rm { = }1}}^{L} {\frac{{S_{{\text{l}}}^{{\text{x}}} }}{{{\text{x}}_{{{\text{n}}\rm { \times }{\text{l}}}}^{{\text{t}}} }}} }}{{\frac{1}{D + V}\left[ {\sum\limits_{{{\text{d}}\rm { = }1}}^{D} {\frac{{S_{{\text{d}}}^{{{\text{y}}^{{\text{g}}} }} }}{{\left( {{\text{y}}_{{{\text{n}}\rm { \times }{\text{d}}}}^{{\text{g}}} } \right)^{{\text{t}}} }}} + \sum\limits_{{{\text{v}}\rm { = }1}}^{V} {\frac{{S_{{\text{v}}}^{{{\text{yb}}}} }}{{\left( {{\text{y}}_{{{\text{n}}\rm { \times }{\text{v}}}}^{{\text{b}}} } \right)^{{\text{t}}} }}} } \right]}} \\ {\text{s}}{\text{.t}}{\text{. x}}_{{{\text{n}}\rm { \times }{\text{l}}}}^{{\text{t}}} & \rm { \ge }\sum\limits_{{{\text{n}} = 1\rm {, }{\text{n}} \ne {0}}}^{N} {{\text{z}}_{n} {\text{x}}_{{{\text{n}}\rm { \times }{\text{l}}}}^{{\text{t}}} \rm {, }l = 1\rm {, } \cdots \rm {, }L} \\ \left( {{\text{y}}_{{{\text{n}}\rm { \times }{\text{d}}}}^{{\text{g}}} } \right)^{{\text{t}}} & \le \sum\limits_{{{\text{n}} = 1\rm {, }{\text{n}} \ne {0}}}^{N} {{\text{z}}_{n} \left( {{\text{y}}_{{{\text{n}}\rm { \times }{\text{d}}}}^{{\text{g}}} } \right)^{{\text{t}}} \rm {, }{\text{d}} = 1\rm {, }...\rm {, }D} \\ \left( {{\text{y}}_{{{\text{n}}\rm { \times }{\text{v}}}}^{{\text{b}}} } \right)^{{\text{t}}} & \rm { \ge }\sum\limits_{{{\text{n}} = 1\rm {, }{\text{n}} \ne {0}}}^{N} {{\text{z}}_{n} \left( {{\text{y}}_{{{\text{n}}\rm { \times }{\text{v}}}}^{{\text{b}}} } \right)^{{\text{t}}} \rm {, }{\text{v}} = 1\rm {, }...\rm {, }V} \\ S_{{\text{l}}}^{{\text{x}}} & \rm { \ge }0\rm {,\, }S_{{\text{d}}}^{{{\text{y}}^{{\text{g}}} }} \rm { \ge }0\rm {,\,}S_{{\text{v}}}^{{{\text{yb}}}} \rm { \ge }0 \\ {\text{z}}_{n} & \rm { \ge 0,\,}\sum\limits_{{{\text{n}} = 1,{\text{n}} \ne {0}}}^{N} {{\text{z}}_{n} = 1} \rm {, }{\text{n}}\rm { = 1, }...\rm {, {\rm N}} \\ \end{aligned}$$

where *ρ* is the target efficiency value, that is, the green efficiency of urban construction land, *ρ* > 0, The larger *p* value, the higher the efficiency value. *xt n* × *l* is the input variable in period *t*, (*yg n* × *d*)^*t*^ is the expected output variable in period *t*, and (*yb n* × *v*)^*t*^ is the unexpected output variable in period *t*; *Sx l* is the relaxation variable of the input factor, *Sy*^*g*^* d* and *Sy*^*b*^* v* are relaxation variables of expected and unexpected outputs, respectively; *z*_*n*_ is the weight vector.

After determining the measurement method of GEUCL, appropriate input indicators, expected output indicators and unexpected output indicators should be selected, so as to accurately measure the efficiency values. By referring to relevant research^21, 23, 25, 42^, land, capital and labor are selected as input factors. In terms of land factor, since the research object is construction land, the area of urban construction land is used to represent the land input. In terms of capital factor, the urban capital stock can reflect the total capital of a city at a certain point, so the urban capital stock is selected to represent the capital input, first, based on the base period of 2003, the provincial fixed asset investment price index is used to convert the nominal fixed asset investment into the actual value, and then the capital stock is calculated using the sustainable inventory method^43^. In terms of labor force factor, urban construction land mainly meets the demand for land factor in the production and operation of the secondary and tertiary industries and the construction of supporting infrastructure. In other words, the number of employees in the secondary and tertiary industries can reflect the input of labor force factors in the utilization of urban construction land. Therefore, the number of employees in the secondary and tertiary industries is selected to measure the labor force input. As for the expected output, based on the fact that urban construction land mainly serves the production and operation of the secondary industry and the tertiary industry, the added value of the secondary and tertiary industries is used as the expected output index, and the regional GDP deflator is used to convert its nominal value into the actual value. As for the unexpected output, because the negative external effect generated during the development and utilization of urban construction land is the damage and pollution to the environment, in this paper, pollutant discharge is regarded as the unexpected output, and the industrial wastewater emissions, industrial smoke and dust emissions, and industrial sulfur dioxide emissions are taken as the unexpected output indicators. The descriptive statistics of input and output indicators are shown in Table 1.

**Table 1:** The descriptive statistics of input and output indicators.

Primary indicator	Secondary indicators	Mean	Std. Dev.	Min	Max
Inputs	Urban construction land area	128.960	265.902	2.340	2915.560
	Capital stock	2.60e + 07	4.91e + 07	1,062,917	5.00e+08
	Number of employees in the secondary and tertiary industries	30.764	62.382	0.770	714.090
Expected output	The added value of the secondary and tertiary industries	7,257,449	1.69e + 07	85,149.660	2.15e+08
Unexpected outputs	Industrial wastewater emissions	8646.251	11,744.120	60	85,735
	Industrial smoke and dust emissions	24,059.020	40,048.530	259	1,347,367
	Industrial SO_2_ emissions	52,836.960	66,808.420	287	683,162

#### Spatial autocorrelation analysis

In this paper, the method of spatial autocorrelation analysis is adopted to explore whether GEUCL in China's Yangtze River Economic Belt has spatial correlation and agglomeration characteristics, and the global Moran’s *I* index is calculated to judge such spatial autocorrelation. The specific formula of global Moran’s *I* index is as follows:

3 $${\text{I}}\rm { = }\frac{{N\sum\limits_{{i\rm { = }1}}^{N} {\sum\limits_{j = 1}^{N} {{\text{w}}_{ij} } \left( {{\text{x}}_{i} - {\overline{\text{x}}}} \right)\left( {{\text{x}}_{{\text{j}}} - {\overline{\text{x}}}} \right)} }}{{\sum\limits_{{i\rm { = }1}}^{N} {\left( {{\text{x}}_{i} - {\overline{\text{x}}}} \right)^{2} } \sum\limits_{{i\rm { = }1}}^{N} {\sum\limits_{j = 1}^{N} {{\text{w}}_{{{\text{ij}}}} } } }}$$

where N is the number of cities, *x*_*i*_, *x*_*j*_ are the GEUCL observations of city *i* and city *j*, respectively, $\overline{x}$ is the average value of the observations. *I*-value ranges from − 1 to 1, if *I* > 0, it indicates that GEUCL has positive spatial correlation, and the closer *I*-value is to 1, the stronger spatial agglomeration is; if *I* < 0, it indicates that GEUCL has negative spatial correlation, and the closer *I*-value is to − 1, the stronger spatial differentiation is; if *I* = 0, it indicates that GEUCL has no spatial autocorrelation, and has random distribution characteristics. In addition, the Z-statistic is used to judge the significance of spatial autocorrelation in this paper, when given∣Z∣ > 1.96, it represents that GEUCL has significantly spatial autocorrelation.

Where *w*_*ij*_ is an element in the spatial weight matrix W, which is used to describe the spatial proximity relationship between cities, this paper does not simply take whether the geographical positions of cities are adjacent, that is, *w*_*ij*_ = 1 when the geographical positions of two cities are adjacent, and *w*_*ij*_ = 0 when they are not adjacent, as the standard for constructing the spatial weight matrix, but adopts other three forms of spatial weight matrix. The first kind of spatial weight matrix is the spatial weight matrix of geographical distance (W^*d*^), and the reciprocal of geographical distance between cities is selected as the element, this matrix considers the influence of geographical distance on the estimation results of spatial econometric model, and also considers the possible interaction between cities with close geographical distance but not adjacent geographical location. The element is defined as follows:

4 $$w_{{ij}}^{d} = \left\{ \begin{gathered} \frac{1}{{d_{{ij}}^{2} }},{\text{ }}if{\text{ }}i \ne j \hfill \\ 0,{\text{ }}if{\text{ }}i{\text{ }} = {\text{ }}j \hfill \\ \end{gathered} \right.$$

where *d*_*ij*_ is the distance between city i and city j calculated through latitude and longitude data of cities.

The second kind of spatial weight matrix is the spatial weight matrix of economic distance (W^*e*^), and the reciprocal of the gap of economic development level between cities is selected as the element, and the gap of resident income between cities is used to measure the gap of economic development level. This matrix extends the distance of geographical location between cities to the gap of economic development level, and considers the impact of the gap of economic development level on the estimation results of spatial econometric model, its element is defined as follows:

5 $$w_{{ij}}^{e} = \left\{ \begin{gathered} \frac{1}{{\left| {PGDP_{i} - PGDP_{j} } \right|}},{\text{ }}if{\text{ }}i \ne j \hfill \\ 0,{\text{ }}if{\text{ }}i{\text{ }} = {\text{ }}j \hfill \\ \end{gathered} \right.$$

where PGDP_*i*_ and PGDP_*j*_ respectively represent the average actual per capita GDP of city *i* and city *j* from 2003 to 2017, and the actual per capita GDP is calculated by the GDP deflator.

The third kind of spatial weight matrix is the nested weight matrix of economy and geography (W^*de*^), and the sum of the weights of geographical distance and economic distance is selected as the element. In reality, the spatial correlation between cities is usually influenced by both geographical distance and economic characteristics, so the spatial weight matrix constructed by integrating the geographical location and economic characteristics of the city is more suitable for estimating the spatial econometric model, and the element is set as follows:

6 $$w_{{ij}}^{{de}} = \left\{ \begin{gathered} \sigma w_{{ij}}^{d} + \left( {1 - \sigma } \right)w_{{ij}}^{e} ,{\text{ }}if{\text{ }}i \ne j \hfill \\ 0,{\text{ }}if{\text{ }}i{\text{ }} = {\text{ }}j \hfill \\ \end{gathered} \right.$$

where *wd ij* is the element in the weight matrix of geographical distance, as shown in Eq. (4), *we ij* is the element in the weight matrix of economic distance, as shown in Eq. (5), σ is the weight of the weight matrix of geographic distance, drawing on related study^44^, this paper sets σ to 0.5.

#### Spatial econometric model

In order to further study the spatial characteristics of GEUCL, this paper constructs a spatial autoregressive model to explore the spatial spillover effects of GEUCL, and investigate whether GEUCL has mutual influence between neighboring cities. The spatial autoregressive model constructed in this paper is as follows:

7 $$UCLGE_{{{\text{it}}}} \rm { = }\beta_{0} { + }\varphi \sum\limits_{{{\text{j}} = 1}}^{N} {{\text{w}}_{{{\text{ij}}}} } UCLGE_{{{\text{jt}}}} + \beta_{1} GO_{it} + \beta_{2} IN_{it} + \beta_{3} HU_{it} + \beta_{4} OP_{it} + \nu_{i} + \delta_{t} + \varepsilon_{it}$$

Where GEUCL represents the green efficiency of urban construction land, and *w*_*ij*_ is the element in the spatial weight matrix, as shown in Eqs. (4)–(6). *φ* is spatial autocorrelation coefficient, that is, the spatial spillover effect between cities, if *φ* > 0, it suggests GEUCL has positive spatial spillover effect; if *φ* < 0, it indicates that GEUCL has negative spatial spillover effect. *β*_*0*_ represents the constant term, *ε* is random disturbance, *ν* is the spatial fixed effects, *δ* represents the temporal fixed effects. Considering there are many factors affecting the green efficiency of urban construction land, this paper introduces a set of control variables, they are government intervention (GO), industrial structure (IN), human capital (HU), the level of opening up (OP), *β*_*1*_, *β*_*2*_, *β*_*3*_, *β*_*4*_ are the coefficients of control variables. Government intervention (GO) is represented by the ratio of the expenditure within general government budget to gross regional product. Industrial structure (IN) is measured by the ratio of added value of tertiary industry to that of secondary industry. Human capital (HU) is measured by the natural logarithm of the number of teachers in colleges and universities. The level of opening up (OP) is represented by the natural logarithm of the amount of foreign capital actually utilized.

In order to further study the spatial spillover effects of GEUCL, based on the perspective of urban hierarchy, this paper refines the analysis of spatial interaction between cities, and explores the spatial spillover effects of GEUCL between cities of different grades. Firstly, according to the urban population of each city, the sample cities are divided into three classes: large cities, medium cities and small cities, those with urban population more than 1 million are defined as large cities, those with urban population between 500,000 and 1 million are defined as medium cities, and those with urban population less than 500,000 are defined as small cities, which are represented by symbols L, M and S respectively. Secondly, the spatial lag variable of GEUCL is set as *GEUCL*^*lag*^, and by referring to relevant research^45, 46^, *GEUCL*^*lag*^ is decomposed into:

8 $$UCLGE^{{{\text{lag}}}} \rm = UCLGE^{{\text{L}}} + UCLGE^{{\text{M}}} + UCLGE^{S}$$

Finally, dummy variables L, M and S used to identify three types of cities are set, which are respectively multiplied with Eq. (8) to obtain spatial lag variables: *GEUCL*^*L*^*L*, *GEUCL*^*L*^*M*, *GEUCL*^*L*^*S*, *GEUCL*^*M*^*L*, *GEUCL*^*M*^*M*, *GEUCL*^*M*^*S*, *GEUCL*^*S*^*L*, *GEUCL*^*S*^*M*, *GEUCL*^*S*^*S*, *GEUCL*^*L*^*L*, *GEUCL*^*L*^*M* and *GEUCL*^*L*^*S*, they can be used to estimate spatial spillover effects between cities of different classes and between cities of the same class, respectively. Therefore, Eq. (7) evolves into the following formula:

9 $$\begin{aligned} UCLGE_{{{\text{it}}}} & \rm = \beta_{0} { + }\varphi_{1} UCLGE_{{{\text{it}}}}^{L} M + \varphi_{2} UCLGE_{{{\text{it}}}}^{L} S + \varphi_{3} UCLGE_{{{\text{it}}}}^{M} S + \varphi_{4} UCLGE_{{{\text{it}}}}^{M} L \\ & \;\; + \varphi_{5} UCLGE_{{{\text{it}}}}^{S} L + \varphi_{6} UCLGE_{{{\text{it}}}}^{S} M{ + }\varphi_{7} UCLGE_{{{\text{it}}}}^{L} L{ + }\varphi_{8} UCLGE_{{{\text{it}}}}^{M} M \\ & \;\; + \varphi_{9} UCLGE_{{{\text{it}}}}^{S} S + \beta_{1} GO_{it} + \beta_{2} IN_{it} + \beta_{3} HU_{it} + \beta_{4} OP_{it} + \nu_{i} + \delta_{t} + \varepsilon_{it} \\ \end{aligned}$$

where *φ*_*1*_, *φ*_*2*_, *φ*_*3*_ are the spatial spillover effects of large cities on medium cities and small cities, and medium cities on small cities, respectively, namely the spatial spillover effects of higher class of cities on lower class of cities; *φ*_*4*_, *φ*_*5*_, *φ*_*6*_ are the spatial spillover effects of medium cities on large cities, and small cities on large cities and medium cities, namely the spatial spillover effects of lower class of cities on higher class of cities; *φ*_*7*_, *φ*_*8*_, *φ*_*9*_ are the spatial spillover effects between large cities, between medium cities, and between small cities.

## Results

### Analysis of temporal variation of GEUCL

**Figure 2:**
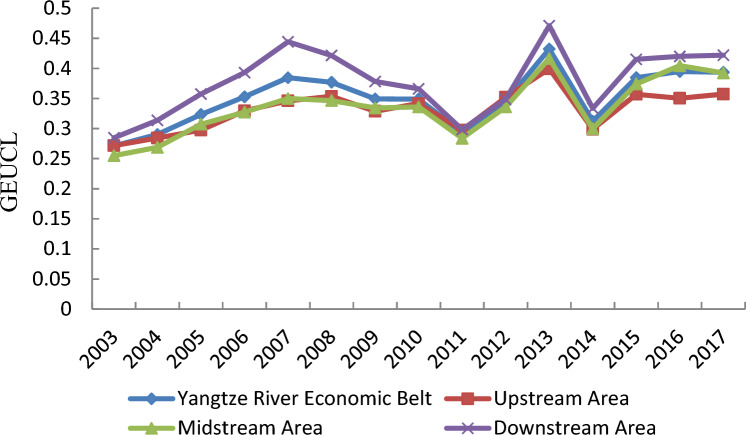
The change trend of average GEUCL in the Yangtze River Economic Belt of China from 2003 to 2017.

Based on the super-efficiency SBM model, this paper uses MaxDEA software to calculate GEUCL of 108 cities in China's Yangtze River Economic Belt. Figure 2 depicts the change trend of average GEUCL in China's Yangtze River Economic Belt from 2003 to 2017. During the study period, the average efficiency value presents a fluctuating upward trend, rising from 0.27 in 2003 to 0.39 in 2017, and the cumulative increase rate is 44.44%, with an average annual growth rate of 3.14%, which represents that the green utilization of urban construction land in the Yangtze River Economic Belt achieves certain results during the study period. However, the average efficiency value is lower, which indicates that the green utilization level of urban construction land still has a large room for improvement.

Specifically, the change of GEUCL in the Yangtze River Economic Belt can be divided into three stages: the first stage is from 2003 to 2011, the second stage is from 2011 to 2014, and the third stage is from 2014 to 2017. In the first stage, the average efficiency value presents an inverted “V” shaped trend, during 2003–2007, the average efficiency value shows a steady rise, rising from 0.27 in 2003 to 0.38 in 2007, however, during 2007–2011, the average efficiency value presents a gradual decline, it falls from 0.38 in 2007 to 0.29 in 2011. In the second stage, the average efficiency value also presents an inverted “V” shaped trend, during 2011–2013, the average efficiency value shows an increasing trend, rising from 0.29 in 2011 to 0.43 in 2013, however, in just one year, the average efficiency value decreases from 0.43 in 2013 to 0.31 in 2014. In the third stage, the average efficiency value shows a gradual upward trend, increasing from 0.31 in 2014 to 0.39 in 2017. The fluctuation of the average efficiency value means that the green utilization of urban construction land in the Yangtze River Economic Belt is still in constant exploration, lack of long-term mechanism, and the sustainable improvement of green use of construction land has not been realized.

Regionally, in the upstream region, the average efficiency value increases by 33.33% from 0.27 to 0.36 during 2003–2017. In the midstream region, the average efficiency value increases by 56% from 0.25 in 2003 to 0.39 in 2017. In the downstream area, the average efficiency value increases by 50% from 0.28 to 0.42 during 2003–2017. In terms of growth rate, during the study period, the growth rate of the average efficiency value in the midstream area is the highest, followed by that in the downstream area, and the lowest in the upstream area, moreover, the growth rates in the midstream and downstream regions are both higher than that in the whole Yangtze River Economic Belt. In terms of absolute value, except 2012, the average efficiency value in the downstream region is higher than that in the whole region and in the upstream and midstream regions in other years. In conclusion, the average efficiency value in the upstream, midstream and downstream regions all show an increasing trend.

### Analysis of spatial distribution characteristics of GEUCL

**Figure 3:**
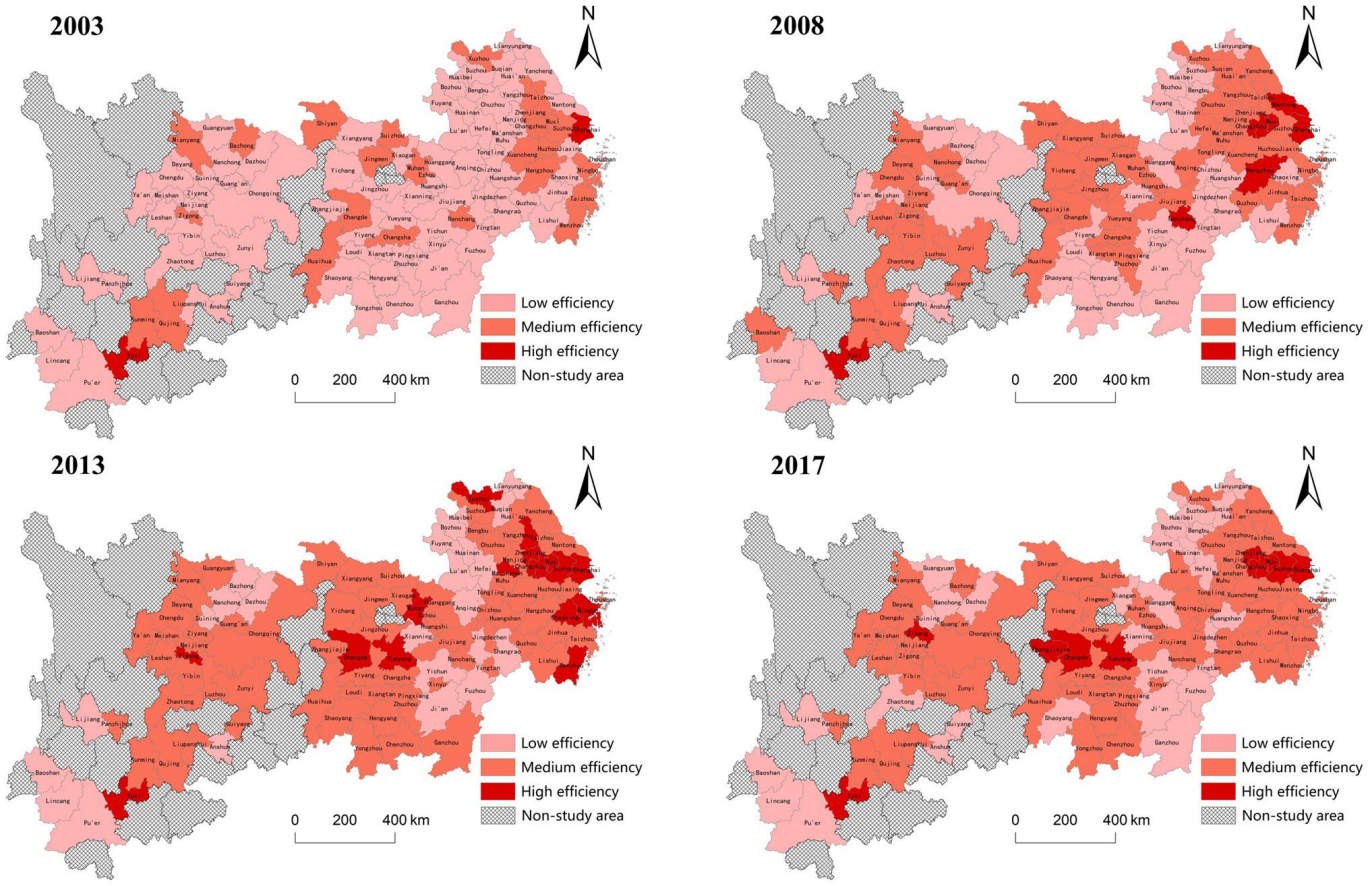
The spatial distribution of GEUCL in the Yangtze River Economic Belt of China.

Based on the measurement results of GEUCL in the Yangtze River Economic Belt, China, and referring to the method of dividing urban land use efficiency that Jin et al. proposed^47^, this paper divides the efficiency value from low to high into three levels. The efficiency value between 0.00 and 0.30 is considered as low efficiency, the efficiency value between 0.30 and 0.60 is considered as medium efficiency, the efficiency value greater than 0.60 is considered as high efficiency. In order to more intuitively reflect the spatial distribution characteristics of GEUCL, the efficiency value was imported into ArcGIS software to draw the spatial distribution map of GEUCL. Figure 3 shows the spatial distribution of GEUCL in the Yangtze River Economic Belt, China in 2003, 2008, 2013 and 2017.

In 2003, there are only 2 high-efficiency cities, which distribute in the upstream and downstream regions, respectively, and few medium-efficiency cities, most of cities are at the low-efficiency level. And the high-efficiency and medium-efficiency cities are scattered and the low-efficiency cities are clustered. In 2008, the number of high-efficiency cities increases to 7, which mostly concentrate in the downstream area, the number of medium-efficiency cities increases significantly to 61, while the number of low-efficiency cities decreases sharply to 40. In general, high-efficiency cities still present scattered distribution, while medium-efficiency cities show concentrated distribution. In 2013, the number of high-efficiency cities increases significantly, reaching 16, and most of them are similarly distributed in the downstream area, and the number of medium-efficiency cities continues to increase to 69, while the number of low-efficiency cities decreases to 23, and the distribution characteristics of medium- efficiency and high-efficiency cities remain unchanged. In 2017, the number of high-efficiency cities declines, leaving only 10, this situation only appears in the downstream region, does not appears in the upstream and midstream regions, and the number of medium-efficiency cities is 67, which remains basically stable, while the number of low-efficiency cities increases to 31, and the distribution characteristics of medium-efficiency and high-efficiency cities still remains unchanged.

All in all, during the study period, the overall efficiency level in the Yangtze River Economic Belt significantly improves, and the number of medium-efficiency and high-efficiency cities increases significantly, while the number of low-efficiency cities decreases sharply. The high-efficiency cities always present scattered distribution, while the medium-efficiency cities change from scattered distribution to agglomeration distribution.

### The examination results of spatial autocorrelation

In this paper, the global Moran’s *I* index calculated by Stata software is used to analyze the spatial autocorrelation of GEUCL in China’s Yangtze River Economic Belt. The global Moran’s *I* index is shown in Table 2, which only lists the results of some years. According to the results in the table, on the basis of the weight matrix of geographical distance, the weight matrix of economic distance and the nested weight matrix of economy and geography, the global Moran’s *I* index in each year is positive and passes the test of the significance level of 1%, which indicates that GEUCL has a significantly positive spatial correlation and presents a spatial agglomeration distribution feature. That is to say, the green efficiency of urban construction land in this city will be affected by the surrounding cities. When the GEUCL of a city is higher, the GEUCL of the city adjacent to its spatial location is also higher.

**Table 2:** The global Moran’s *I* index of GEUCL from 2003 to 2017.

W	2003	2008	2013	2017
W^d^	0.100***	0.149***	0.196***	0.138***
	(3.674)	(4.233)	(5.091)	(3.949)
W^e^	0.108***	0.223***	0.388***	0.153***
	(2.023)	(3.207)	(5.103)	(2.239)
W^de^	0.105***	0.184***	0.270***	0.148***
	(3.872)	(5.208)	(7.014)	(4.266)

Note: the values in parentheses are Z-values; ***p < 0.01, **p < 0.05, *p < 0.1.

### Analysis of spatial econometric results

The above results of spatial autocorrelation analysis only show that GEUCL have spatial autocorrelation, but cannot confirm whether GEUCL will overflow from each other between adjacent cities. Therefore, it is necessary to perform regression on Formula (9) to verify the spatial spillover effect of GEUCL. In order to ensure the robustness of the estimation results, this paper carries out row standardization and maximum eigenvalue standardization on the weight matrix of geographical distance respectively, and also use the weight matrix of economic distance and the nested weight matrix of economy and geography for comparative analysis. In terms of estimation methods, maximum likelihood method is used to estimate Formula (9). Table 3 shows the estimation results. Column (1) is the estimated results based on the weight matrix of geographical distance of row standardization. From the perspective of the spatial spillover effects of higher-grade cities on lower-grade cities, the estimation coefficient of *GEUCL*^*L*^*M* is significantly positive at the 1% level, indicating that the increase of GEUCL in large cities has a significantly positive spatial spillover effect on that in adjacent medium cities. The estimation coefficient of *GEUCL*^*L*^*S* is significantly positive at the level of 10%, representing that the increase of GEUCL in large cities has a significantly positive spatial spillover effect on that in neighboring small cities. The estimated coefficient of *GEUCL*^*M*^*S* is significantly positive at the 1% level, which means that the increase of GEUCL in medium cities has a significantly positive spatial spillover effect on that in neighboring small cities. In conclusion, the increase of GEUCL in higher-grade cities has significantly positive spatial spillover effects on that in adjacent lower-grade cities.

**Table 3:** The estimation results of spatial spillover effects.

Variable	(1)	(2)	(3)	(4)
	W^d^(R)	W^d^(E)	W^e^	W^de^
GO	− 0.008	0.064	0.072	0.033
	(0.056)	(0.055)	(0.054)	(0.044)
IN	0.032	0.046	0.038	0.029
	(0.044)	(0.042)	(0.033)	(0.033)
Hu	0.017**	0.018***	0.020**	0.012
	(0.008)	(0.007)	(0.008)	(0.008)
OP	0.002	0.004	0.004	0.002
	(0.004)	(0.003)	(0.003)	(0.003)
Higher → Lower				
GEUCL^L^M	1.367***	2.380***	0.974***	1.351***
	(0.389)	(0.796)	(0.206)	(0.273)
GEUCL^L^S	0.938*	1.343*	0.549***	0.709*
	(0.536)	(0.710)	(0.150)	(0.415)
GEUCL^M^S	0.941***	0.376	0.979***	1.667***
	(0.245)	(0.517)	(0.189)	(0.273)
Lower → Higher				
GEUCL^M^L	0.809***	0.562**	0.562***	1.023***
	(0.296)	(0.282)	(0.182)	(0.247)
GEUCL^S^L	0.621**	2.322***	0.514*	0.645***
	(0.287)	(0.547)	(0.311)	(0.193)
GEUCL^S^M	1.056***	1.885***	0.446***	0.807***
	(0.156)	(0.536)	(0.142)	(0.153)
Same grade				
GEUCL^L^L	1.471***	1.941***	0.664***	1.029***
	(0.383)	(0.631)	(0.120)	(0.124)
GEUCL^M^M	0.370*	0.040	0.674*	0.681***
	(0.204)	(0.515)	(0.395)	(0.226)
GEUCL^S^S	0.610**	2.262***	0.378**	0.339
	(0.260)	(0.511)	(0.148)	(0.217)
Constant	− 0.142	− 0.107	− 0.109	− 0.105
	(0.090)	(0.082)	(0.069)	(0.082)
Log L	1661.56	1615.38	1629.95	1696.60
N	1620	1620	1620	1620

Note: the values in the parentheses are standard errors; ***p < 0.01, **p < 0.05, *p < 0.1; Log L is Log likelihood; W^d^(R) represents the weight matrix of geographical distance of maximum eigenvalue standardization, W^d^(E) represents the weight matrix of geographical distance of row standardization.

From the perspective of the spatial spillover effects of lower-grade cities on higher-grade cities, the coefficient of *GEUCL*^*M*^*L* is significantly positive at the 1% level, which means that the increase of GEUCL in medium cities has a significantly positive spatial spillover effect on that in neighboring large cities. The coefficient of *GEUCL*^*S*^*L* is significantly positive at the 5% level, which confirms that the increase of GEUCL in small cities has a significantly positive spatial spillover effect on that in adjacent large cities. The coefficient of *GEUCL*^*S*^*M* is significantly positive at the 1% level, indicating that the increase of GEUCL in small cities has a significantly positive spatial spillover effect on that in neighboring medium cities. In conclusion, the increase of GEUCL in lower-grade cities has significantly positive spatial spillover effects on that in neighboring higher-grade cities.

From the perspective of the spatial spillover effects between cities of the same grade, the coefficient of *GEUCL*^*L*^*L* is significantly positive at the 1% level, indicating that there is a significantly positive spatial spillover effect of GEUCL between neighboring large cities. The coefficient of *GEUCL*^*M*^*M* is significantly positive at the 10% level, which means that GEUCL has a significantly positive spatial spillover effect between adjacent medium cities. The coefficient of *GEUCL*^*S*^*S* is significantly positive at the 5% level, representing that GEUCL has a significantly positive spatial spillover effect between neighboring small cities. In conclusion, GEUCL has significantly positive spatial spillover effects between adjacent cities of the same grade. To sum up, GEUCL has significantly positive spatial spillover effects between neighboring cities of different grades and between neighboring cities of the same grade.

Column (2) represents the estimation results based on the weight matrix of geographical distance of maximum eigenvalue standardization, it can be seen that, except the coefficients of *GEUCL*^*M*^*S* and *GEUCL*^*M*^*M*, the other results are consistent with the results in Column (1), indicating that the estimation results based on the weight matrix of geographical distance is robust to a large extent. Column (3) and column (4) report the estimation results based on the weight matrix of economic distance and the nested weight matrix of economy and geography, respectively, it can be found that, except the estimation coefficients of *GEUCL*^*S*^*S* in column (4), the remaining coefficients are significantly positive, indicating that the replacement of the spatial weight matrix does not change the estimation results of the spatial econometric model.

### Regional heterogeneity analysis

The Yangtze River Economic Belt spans the eastern, central and western regions of China with a vast territory, according to the characteristics of the Yangtze River channel and the topography of the basin, it can be divided into upstream area, midstream area and downstream area. These three regions have obvious differences in resource endowment, industrial structure, economic development level and other aspects, which will result in different characteristics of green utilization of urban construction land in each region. Therefore, it is necessary to analyze the regional heterogeneity of spatial spillover effects of GEUCL. In this paper, the samples are divided into upstream area samples, midstream area samples and downstream area samples, and regression analysis is carried out respectively. The estimated results are shown in Table 4. Column (1), column (2) and column (3) are the estimated results of spatial spillover effects in the upstream region, midstream region and downstream region of the Yangtze River Economic Belt, respectively. It can be seen from the results that, in the downstream area, except the spatial spillover effect of small cities on neighboring medium cities, and the spatial spillover effect between adjacent medium cities, the other spatial spillover effects between adjacent cities of different grades and between adjacent cities of the same grade are significantly positive, which is not much different from the estimation results of the whole sample. However, in the upstream and midstream regions, most of the spatial spillover effects are insignificant. The reason may be that the economy in the downstream region of the Yangtze River Economic Belt is relatively developed, the regional economy has achieved coordinated development, and the green utilization of urban construction land has learning effect and imitation effect between cities.

**Table 4:** Regional heterogeneity test results.

Variable	(1)	(2)	(3)
	Upstream area	Midstream area	Downstream area
GO	− 0.115	0.065	− 0.028
	(0.119)	(0.138)	(0.067)
IN	− 0.050	0.134	0.008
	(0.050)	(0.087)	(0.031)
Hu	0.010	0.015	0.009
	(0.015)	(0.015)	(0.023)
OP	− 0.001	0.018	0.000
	(0.003)	(0.012)	(0.007)
Higher → Lower			
GEUCL^L^M	0.521	0.584	1.666**
	(1.619)	(0.387)	(0.666)
GEUCL^L^S	4.045*	0.118	0.654*
	(2.067)	(0.459)	(0.360)
GEUCL^M^S	0.532	1.332***	0.810**
	(0.432)	(0.307)	(0.344)
Lower → Higher			
GEUCL^M^L	4.733**	0.537	0.723**
	(2.024)	(2.240)	(0.283)
GEUCL^S^L	0.275	0.340	0.845**
	(0.950)	(1.704)	(0.403)
GEUCL^S^M	1.056***	0.907*	0.327
	(0.406)	(0.476)	(1.046)
Same grade			
GEUCL^L^L	− 5.096	5.153	1.565***
	(4.210)	(7.176)	(0.430)
GEUCL^M^M	0.775	0.992*	0.363
	(1.579)	(0.561)	(0.390)
GEUCL^S^S	0.089	0.323	0.994***
	(0.610)	(0.288)	(0.208)
Constant	0.004	− 0.371*	− 0.057
	(0.152)	(0.205)	(0.131)
Log L	461.84	580.98	687.69
N	465	540	615

Note: the values in the parentheses are standard errors; ***p < 0.01, **p < 0.05, *p < 0.1; Log L is Log likelihood.

## Discussion

This paper finds that in the Yangtze River Economic Belt of China, GEUCL has significantly positive spatial spillover effects between adjacent cities of different grades and between adjacent cities of the same grade. Among them, the increase of GEUCL in higher-grade cities has significantly positive spatial spillover effects on that in neighboring lower-grade cities, which may be because there is a top-down radiation driving effect of green utilization of urban construction land among cities in the Yangtze River Economic Belt of China, and the achievements of higher-grade cities in green development will have an incentive effect on lower-grade cities, promoting the improvement of GEUCL in lower-grade cities. The increase of GEUCL in lower-grade cities has significantly positive spatial spillover effects on that in neighboring higher-grade cities, which may be because the promotion of green use of construction land in lower-grade cities in China’s Yangtze River Economic Belt will exert pressure on neighboring higher-grade cities, forcing them to adopt relevant policies and measures to improve GEUCL. GEUCL has significantly positive spatial spillover effects between adjacent cities of the same grade, which may be due to the competitive imitation and learning imitation between adjacent cities of the same grade in the Yangtze River Economic Belt. Competitive imitation refers to the behavior of cities imitating their competitors in order to gain a favorable position in the competition. Learning imitation refers to the behavior of cities accumulating experience and avoiding risks through learning, communication and imitation. Since the reform and opening up, motivated by political promotion competitions, in order to promote economic growth, city governments have regarded cities with similar economic development level as competitors or objects of learning, and imitated them in terms of land use policies, industrial policies, and tax policies. The level of economic development among neighboring cities of the same grade is similar, so it is easy to regard each other as a competitor or object of learning, and imitate each other in terms of land use policies, resulting in the convergence of GEUCL among neighboring cities of the same grade, with significantly positive spatial spillover effect.

The conclusions of this paper have certain policy implications. Firstly, establish a land use cooperation mechanism between neighboring cities. The governments of neighboring cities should establish land use cooperation mechanisms by jointly formulating utilization policies of construction land, as well as industrial access standards and environmental protection standards, and sharing the data of land green utilization. At the same time, effectively play the role of regulation of land use policy, promote the green and low-carbon use of construction land, and maximize the spillover effects between neighboring cities, so as to jointly promote the improvement of regional GEUCL. Second, carry out joint rectification of the irrational use of land between neighboring cities. According to the city's functional positioning and specific development stage, neighboring cities jointly formulate land irrational use standards such as pollution discharge and extensive utilization, and carry out joint remediation of land irrational use, so as to reduce environmental pollution and resource waste in the process of land use, optimize land use and industrial structure, and achieve the purpose of green collaborative utilization of regional urban construction land. Third, establish an assessment mechanism for the green efficiency of urban construction land. Take GEUCL as the assessment index of urban governments in promoting green development, and establish a reward and punishment system, at the same time, at the same time, take the assessment index as an important basis for the allocation of construction land indicator. By applying assessment pressure, force urban governments to pay attention to the green utilization of construction land, strictly implement environmental protection policies, and increase support for enterprise technology research and development and green technology transformation, so as to promote the improvement of green efficiency and intensive utilization level of urban construction land.

It should be noted that the research in this paper still has some shortcomings, for example, in the measurement of GEUCL, the construction of the index system is not perfect because some data are not available. In this paper, China's Yangtze River Economic Belt is taken as the research area, and other regions can be taken as the research object in the future to verify whether the research conclusions of this paper are applicable in other regions, and to investigate whether the spatial–temporal evolution law and spatial externalities of GEUCL show different characteristics in other regions. This paper only studies the spatial–temporal evolution and spatial spillover effects of GEUCL, in the future, we can further explore the specific mechanism causing such spatial–temporal evolution characteristics and spatial spillover effects. In addition, we can study influencing factors of GEUCL to form a systematic research system, so as to provide a more solid theoretical foundation for promoting the green utilization of urban construction land and promoting urban sustainable development.

## Conclusions

Based on the panel data of 108 prefecture-level and above cities in the Yangtze River Economic Belt, China from 2003 to 2017, this paper measures the green efficiency of urban construction land and analyzes its spatial–temporal evolution characteristics, in terms of temporal variation, the average efficiency value shows a fluctuating upward trend during the study period, rising from 0.27 in 2003 to 0.39 in 2017, the cumulative growth rate is 44.44%, with an average annual growth rate of 3.14%. In terms of spatial distribution characteristics, during the study period, the number of medium-efficiency and high-efficiency cities increases significantly, while the number of low-efficiency cities decreases sharply; high-efficiency cities always present scattered distribution, while medium-efficiency cities change from scattered distribution to agglomeration distribution.

On the basis of the measurement results of GEUCL, this paper constructs a spatial autoregressive model to study the spatial spillover effects of GEUCL between cities of different grades, and finds that from the perspective of the spatial spillover effects of higher-grade cities on lower-grade cities, the increase of GEUCL in large cities has significantly positive spatial spillover effects on that in adjacent medium cities and small cities, and the increase of GEUCL in medium cities has a significantly positive spatial spillover effect on that in neighboring small cities, that is, the increase of GEUCL in higher-grade cities has significantly positive spatial spillover effects on that in adjacent lower-grade cities. From the perspective of the spatial spillover effects of lower-grade cities on higher-grade cities, the increase of GEUCL in medium cities has a significantly positive spatial spillover effect on that in neighboring large cities, and the increase of GEUCL in small cities has significantly positive spatial spillover effects on that in adjacent large cities and medium cities, that is, the increase of GEUCL in lower-grade cities has significantly positive spatial spillover effects on that in neighboring higher-grade cities. From the perspective of the spatial spillover effects between cities of the same grade, there are significantly positive spatial spillover effects of GEUCL between neighboring large cities, between neighboring medium cities and between neighboring small cities, that is, GEUCL has significantly positive spatial spillover effects between adjacent cities of the same grade. To sum up, GEUCL has significantly positive spatial spillover effects between neighboring cities of different grades and between neighboring cities of the same grade.

Through regional heterogeneity analysis, this paper finds that the spatial spillover effects of GEUCL are characterized by heterogeneity in the upstream, midstream and downstream regions of the Yangtze River Economic Belt. In the downstream region, the estimation results are not much different from the ones of the whole sample. However, in the upstream and midstream regions, the spatial spillover effects of GEUCL are not significant in most cases.

## Data Availability

The datasets used and/or analysed during the current study are available from the corresponding author on reasonable request.
